# Liposarcoma of the Thyroid

**DOI:** 10.1080/1357-7140400001517

**Published:** 2004

**Authors:** A. Mitra, C. Fisher, P. Rhys-Evans, C. Harmer

**Affiliations:** 1 St. Thomas' and Guys' Cancer Centre Lambeth Palace Road London UK; 2 Thyroid and Sarcoma Units Royal Marsden Hospital London UK; 3 Meyerstein Institute Of Oncology The Middlesex Hospital Mortimer Street London W1T 3AA UK

## Abstract

Liposarcoma of the thyroid gland is rare with only 3 cases reported in the English literature. We present a further two patients whom we have recently treated: a 49 year old lady with a myxoid liposarcoma and a 71 year old man with a pleomorphic liposarcoma. Both underwent macroscopic excision of tumour but had positive margins, so were then treated with external beam radiotherapy. The former patient died from metastases 10 months after presentation, the latter remains alive but has developed metastatic disease on follow up at 24 months.

We recommend the use of high dose radiotherapy following radical surgery as margins of excision are usually narrow in this most difficult region. The role of chemotherapy is yet to be established.


*Sarcoma*, June/September 2004, *VOL. 8, NO. 2/3, 91–96*

Taylor & Francis healthsciences

**TWO CASE HISTORIES AND REVIEW**

### Liposarcoma of the thyroid

A. MITRA[1], C. FISHER[2], P. RHYS-EVANS[2], & C. HARMER[2]

[1]*St. Thomas' and Guys' Cancer Centre, Lambeth Palace Road, London and* [2]*Thyroid and Sarcoma Units, Royal Marsden Hospital, London*

**Abstract**

Liposarcoma of the thyroid gland is rare with only 3 cases reported in the English literature. We present a further two patients whom we have recently treated: a 49 year old lady with a myxoid liposarcoma and a 71 year old man with a pleomorphic liposarcoma. Both underwent macroscopic excision of tumour but had positive margins, so were then treated with external beam radiotherapy. The former patient died from metastases 10 months after presentation, the latter remains alive but has developed metastatic disease on follow up at 24 months.

We recommend the use of high dose radiotherapy following radical surgery as margins of excision are usually narrow in this most difficult region. The role of chemotherapy is yet to be established.

**Key words:** *liposarcoma, thyroid*

### Introduction

Cancer of the thyroid gland is rare, accounting for only 1% of all malignancies. Classified as either epithelial or non-epithelial, the majority are of papillary and follicular cell origin, accounting for 95%. Non-epithelial tumours include malignant lymphoma and tumours arising from mesenchymal elements.[1] Our literature search using MEDLINE limited to publications in English, revealed less than 50 primary sarcomas of the thyroid. There have been a few reports of sarcoma metastasising to the thyroid gland ("unpublished observations").

Liposarcoma of the thyroid gland is extremely rare. We found three cases in the English reported literature, one of which was radiation induced. We present a further two cases and discuss the ideal management of this uncommon condition.

### Case History 1

A 49-year-old Greek Cypriot woman presented with a one-year history of an enlarging swelling in her neck, associated with 3 months increasing shortness of breath and dysphagia. She had a large, fixed, hard mass in the thyroid gland with retrosternal extension. Fifteen years earlier, she had undergone a partial thyroidectomy for a multi-nodular goitre but this histology was not available for review.

Tru-cut biopsy gave the diagnosis of myxoid liposarcoma. Computerised tomography scan revealed a 7 cm mixed attenuation soft tissue mass within the right lobe of the thyroid, displacing and compressing both trachea and oesophagus. The mass appeared inseparable from the right carotid artery and internal jugular vein, encasing the carotid artery and extending through the thoracic inlet to the origin of the right main bronchus (Fig. 1). The patient deteriorated developing neck pain and stridor.

Bronchoscopy showed the right vocal cord was not functioning. There was severe compression of the trachea along its entire length, narrowing its lumen to a crescent. At surgery, there was a multinodular mass in the right lobe of the thyroid, measuring $12 \times 7 \times 5$ cm, adherent to the wall of the trachea and infiltrating the pharyngeal muscle. Following division of the strap muscles and the right recurrent laryngeal nerve, resection of the tumour was macroscopically complete.

Histological examination demonstrated a malignant neoplasm diffusely infiltrating the thyroid parenchyma, adjacent soft tissues and skeletal muscle. The tumour comprised a mixture of elements ranging from highly pleomorphic giant cells to

Correspondence to: Dr. A. Mitra, Meyerstein Institute Of Oncology, The Middlesex Hospital, Mortimer Street, London, W1T 3AA, UK. E-mail: anitamitra@doctors.org.uk

1357-714X print/1369–1643 online © 2004 Taylor & Francis Ltd
DOI: 10.1080/1357-7140400001517

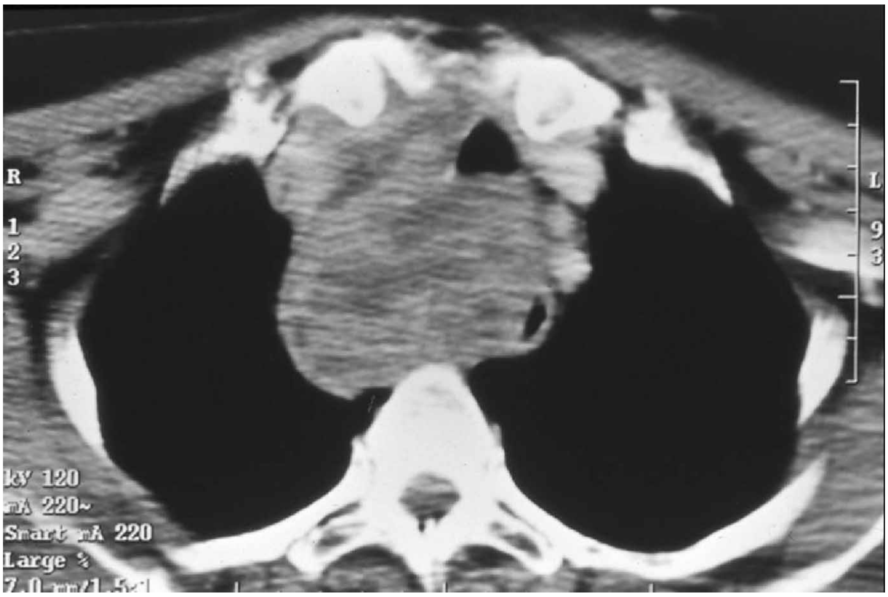

**Fig. 1.** *Computerised tomography scan showing a mixed attenuation soft tissue mass within the right lobe of the thyroid.*

mildly pleomorphic, irregular, spindle-shaped nuclei. The highest mitotic rate was 10 per 10 hpf, EORTC grade 2. The stroma was markedly myxoid and there was an arborising capillary vasculature. Tumour extended to the resection margins.

Four weeks following surgery, external beam radiotherapy to the neck and superior mediastinum completed treatment. This comprised a two-phase technique. Anterior and undercouched fields covered both sides of the neck and superior mediastinum in phase 1 to a dose of 28 Gy in 14 fractions treated daily. Phase 2 encompassed the tumour bed and received 30 Gy in 15 fractions daily, to a CT planned volume. During treatment, the patient developed a chest infection and experienced oesophagitis requiring a short admission, causing reduction of the phase 1 dose and extension of the total treatment time.

She remained well until 7 months after radiotherapy when she developed subcutaneous nodules in her scalp, weight loss and abdominal pain. The primary site remained free of disease. CT scan revealed lung and liver metastases. She received single agent doxorubicin at 60 mg/m$^2$. The liver disease progressed after one cycle and Ifosfamide at 2.5 g/m$^2$ was substituted. After one cycle, she developed renal failure. Further chemotherapy would have been inappropriate and she returned to Cyprus where she died 10 months after her original presentation.

### Case History 2

A 71-year-old retired man presented with a one-month history of difficulty in breathing, a feeling of constriction in his neck and hoarseness of the voice. These symptoms progressed to marked dysphagia and deafness in the left ear.

On examination, he had mild stridor and a left secretory otitis media. Endoscopic examination of his posterior nasal space was unremarkable. Palpation of his neck revealed a firm mass above the sternal notch, more prominent to the left of the midline, which did not move on swallowing. There was no lymphadenopathy and flexible laryngoscopy showed both vocal cords to be mobile. Computerised tomography scan confirmed a mass arising from the left lobe of the thyroid, extending from the thoracic inlet behind the brachiocephalic vein to the level of the aortic arch.

His symptoms progressed rapidly although steroids relieved the stridor. Fine needle aspiration cytology was not diagnostic. Open thyroid biopsy was undertaken and the middle ear effusion drained. The thyroid tissue was slightly nodular with mild variation in follicle size; there was mild focal inflammation in the capsule, but no features of malignancy. A biopsy taken from the left side of the post-nasal space showed a moderate chronic inflammatory infiltrate, but no features of malignancy.

Surgical exploration was required to relieve the tracheal compression. Bronchoscopy revealed an oedematous larynx and severe compression of the cervical and upper thoracic trachea. Tissues of the neck were compressed and the oesophagus was adherent to the tumour. After division of the strap muscles, the medial aspect of the gland was mobilised and left hemi-thyroidectomy was carried out. Excision was macroscopically complete.

Histology confirmed a pleomorphic liposarcoma extending to the margins of excision (Fig. 2). Post-operative recovery was complicated by the

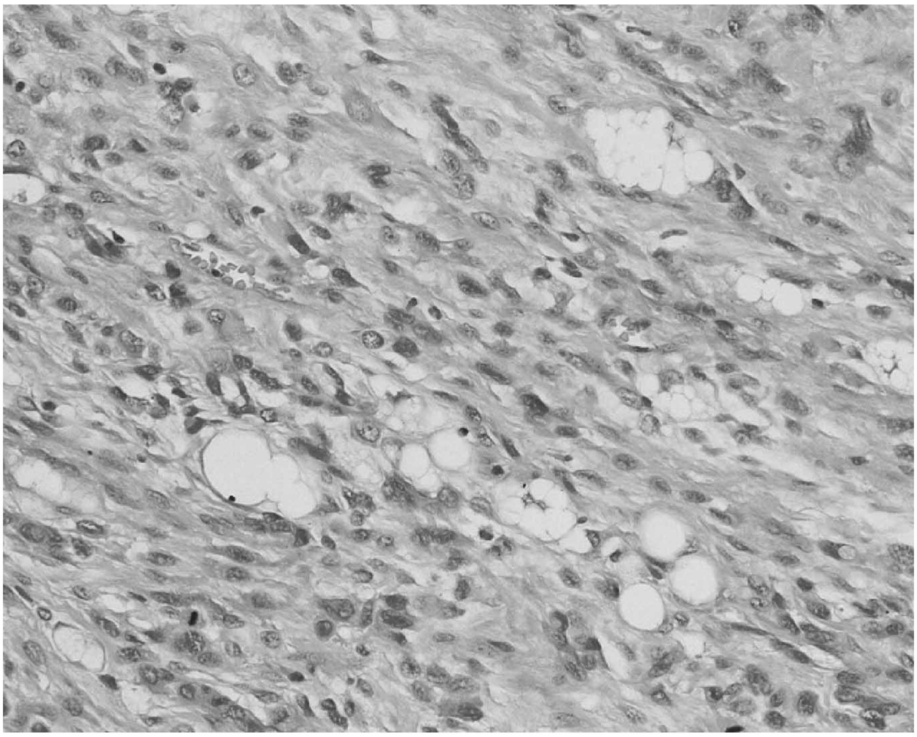

**Fig. 2.** *Pleomorphic liposarcoma.*

development of a deep vein thrombosis. He was prescribed replacement thyroxine and CT scan excluded pulmonary metastases.

Radical dose radiotherapy completed his treatment. Phase 1 utilised anterior and undercouched fields encompassing both sides of the neck and superior mediastinum, delivering a mid-plane dose of 46 Gy in 23 daily fractions. Phase 2 encompassed the original tumour volume to 14 Gy in 7 fractions, bringing the total dose to 60 Gy over 6 weeks.

Follow up at 11 months found him asymptomatic with no evidence of local recurrence but routine chest x-ray revealed multiple and bilateral unresectable lung metastases. Single agent doxorubicin, 60 mg/m$^2$, was given for 3 cycles with the second complicated by admission for neutropenic sepsis. In the absence of response no further chemotherapy was administered. He remains asymptomatic from his lung disease 14 months after their diagnosis but has developed bone metastases; right acetabular involvement requiring palliative radiotherapy.

### Discussion

Liposarcoma forms a subset of the rare diagnosis of non-epithelial thyroid malignancies. Other types include primary thyroid lymphoma, forming 2% of extra-nodal lymphomas, malignant teratoma and primary squamous cell carcinoma.[2] Isolated case reports of thyroid sarcoma exist and include fibrosarcoma, leiomyosarcoma, osteosarcoma, chondrosarcoma and malignant diffuse haemangiopericytoma.[3] There are also reports of liposarcoma metastasing to the thyroid gland.

The peak incidence of liposarcoma, the second commonest variety of soft tissue sarcoma, is in the 5th and 6th decades and classically occurs in the extremities and retroperitoneum. With a male predominance, its aetiology and pathogenesis are unknown. It is classified into well-differentiated, myxoid, round cell, pleomorphic and dedifferentiated types. The well-differentiated type is relatively indolent and has a high rate of local recurrence but rarely metastasises. The myxoid and round cell types are slightly more aggressive. These types have a 75–100% 5-year survival. Pleomorphic cell types have a higher rate of local recurrence and are more likely to result in distant metastases, with a five-year survival of 20%. The dedifferentiated type contains both the well-differentiated type and a non-lipogenic sarcoma resembling a malignant fibrous histiocytoma or a pleomorphic fibrosarcoma. Behaviour probably depends on the amount of dedifferentiation.[4,5]

Undifferentiated (anaplastic) carcinomas of the thyroid resemble sarcomas to such a degree that differential diagnosis is at best difficult and sometimes impossible. Most malignant thyroid tumours with a sarcoma-like appearance show signs of epithelial differentiation by morphologic or immunohistochemical criteria and are therefore classified as undifferentiated carcinoma. As undifferentiated thyroid carcinomas are exceptionally rare before the age of 50, the possibility of a sarcoma-like thyroid tumour actually being a true sarcoma increases as the patient's age decreases.[6]

Lipoblasts do not necessarily indicate a diagnosis of liposarcoma. They are often found in myxoid and sclerosing liposarcomas but are not essential for these two diagnoses. They are however essential for the diagnosis of the pleomorphic and round cell liposarcoma. Immunohistochemistry staining positive for S100 protein may be helpful in differentiating a true sarcoma although if strongly positive; may be suggestive of melanoma or epitheloid malignant peripheral nerve sheath tumour. CD68 can be positive in some liposarcomas.[7]

We have presented the fourth and fifth cases of thyroid liposarcoma in the English reported literature. The first was a 56-year-old woman with a myxoid liposarcoma and a history of goitre. She initially refused surgery but consented on developing dyspnoea. Her condition deteriorated post-operatively and she died 2 months later of an unspecified cause.[3] The second described an elderly man who, three years after the diagnosis of a presumed thyroid adenoma, developed hoarseness and dysphagia as the thyroid rapidly enlarged. There was no post-operative treatment following removal of a well-differentiated thyroid liposarcoma and he died 2 years later from pneumonia unrelated to disease progression.[8] The third, was a case of a radiation induced myxoid liposarcoma in a 23-year-old man, who presented 12 years after receiving high dose radiotherapy for a T2N1 lymphoepithelioma of the nasopharynx. After surgery and brachytherapy, he remained well on follow up at 22 months.[9]

As with sarcomas at any site, surgery remains the definitive and potentially curative treatment. The likelihood of microscopic disease invading surrounding tissues and the possibility of skip lesions mean that wide margins are required for adequate resection. This is impossible in thyroid sarcomas and in head and neck sarcomas in general, since at presentation the majority of tumours have extended beyond the capsule and lie in close proximity to vital structures. Wide functional resection remains the objective.

Referral to specialist sarcoma unit and detailed pre-operative imaging permits the probability of maximal resection of the tumour at the first operation. If in the rare circumstances where surgery has not been maximally cytoreductive, we would recommend further resection if operable macroscopic disease remains. For thyroid sarcoma, total, or near total thyroidectomy would be ideal. However, as in our second case, it may not be worth second operation to total thyroidectomy in the event of complete macroscopic excision.

Survival in head and neck sarcomas is less than that of limb sarcomas and local recurrence is more frequent. A series of 130 head and neck sarcomas treated at the Royal Marsden Hospital showed an overall 5-year survival of only 50%. Results with surgery alone were poor as were those with radiotherapy as sole treatment. Surgery with radiotherapy, however, improved survival.[10] Radiotherapy is recommended pre- or post-operatively in curative management.

The use of radiotherapy alone in thyroid liposarcoma is not recommended except for cases where poor patient performance status precludes surgery. The relative radioresistance of these tumours demands high dose. Even if only palliation of potential fungation or haemorrhage is the aim. As our second case shows, in the presence of indolent disease, local control is of paramount importance. Patients may succumb to metastatic disease before local recurrence develops.

Vital structures adjacent to the thyroid mean a high degree of precision is required. With conventional radiotherapy, a shrinking field technique is used to minimise the high dose volume. As for sarcomas at other sites, the phase 1 volume should encompass all planes of potential tumour spread. As nodal spread is not a feature, encompassing neck nodes is not necessary. Phase 1 should receive a dose of 46 Gy in 2 Gy daily fractions, to remain within spinal cord tolerance, with margins of 5 cm around the original gross tumour volume. Phase 2 delivers an additional 14 Gy in 2 Gy daily fractions, with a margin of 2 cm, avoiding spinal cord. The phase 2 volume is CT planned with a multi-leaf collimator and requires a perspex shell for accurate positioning. A third phase can be given to 66 Gy if positive margins are documented.

Early radiation oesophagitis though transient may lead a poor tolerance of higher doses of radiation. Late radiation morbidity also manifesting as dysphagia precludes a dose of greater than 60 Gy in most situations. Failure to complete the primary peristaltic wave has been demonstrated by dynamic studies after doses of 45–60 Gy.[11] When compared to conventional radiotherapy, three-dimensional conformal radiotherapy enables a reduction of the amount of normal tissue irradiated and thus complications of dysphagia and myelitis, although care should be taken to ensure adequate coverage of the tumour volume. The advent of Intensity Modulated Radiotherapy (IMRT) may allow reduced normal tissue damage, along with improved coverage of the planned target volume and dose escalation is theoretically possible but will still be limited by oesophageal tolerance.[12]

The energy range should be between 4 and 8 mega-volt photons. A direct electron beam may be used for the last few fractions of radiotherapy but is usually sub-optimal for delivering the greater part of the dose, which should be given by at least two, possibly three, complementary fields. The whole length of the scar should lie within the phase 1 volume. Recurrence is more likely to occur at depth and thus it is unnecessary to use build-up to bring the maximum dose to the surface.[13]

As radiotherapy is often a crucial component in the effective treatment of sarcoma, ways of increasing therapeutic gain have been investigated. Pre-operative radiotherapy was compared to post-operative radiotherapy at the Princess Margaret Hospital. Their randomised trial showed an increase in wound complications in the pre-operative group.[14] There are no randomised trials comparing survival, although Suit *et al.* did show benefit in local control in one series of patients published in 1981.[15] Other more recent trials have shown no benefit.[16] We recommend surgery first, wherever possible, as definitive treatment. No significant improvement in overall results has been observed with brachytherapy over conventional external beam radiotherapy.[16] Hyperfractionated radiotherapy with the aim of delivering a higher total dose of radiation without an increase in late normal tissue damage has shown to be a feasible regime with comparable local control rates. Randomised studies comparing it to conventional treatment are awaited.[17]

Chemotherapy for sarcoma is indicated for symptomatic disease that may be either metastatic or recurrent tumour at the primary site in patients previously treated by maximal surgery and radiotherapy. In high risk patients it can be used as adjuvant or neoadjuvant treatment but this is not yet of proven survival benefit. A few studies show an improvement in local recurrence rates. A meta-analysis of 14 trials of doxorubicin-based chemotherapy published in 1997 had a median follow up of 9.4 years and showed a significantly improved time to local and distant recurrence. Overall survival whilst improving at 10 years from 50–54% was not statistically significant.[18] The largest single study of adjuvant chemotherapy in soft tissue sarcoma was performed by the European organisation for research and treatment of cancer.[19] 468 patients were treated with surgery for their primary sarcoma, and with adjuvant radiation used for margins less than 1 cm. Patients were randomised to receive or not receive combination chemotherapy with cyclophosphamide, vincristine, doxorubicin and dacarbazine given every 28 days for 8 cycles. Disease free survival and local control were both better in the chemotherapy arm but overall survival was not significantly different between the 2 arms. The accrual time, however, was 11years and nearly half the patients were unable to complete the chemotherapy. A relatively large number of patients were ineligible for analysis, most commonly due to inappropriate radiotherapy.

Few cytotoxic agents are active for the treatment of soft tissue sarcomas. Those with activity include doxorubicin, singly or in combination with ifosfamide and occasionally dacarbazine. Response rates are in the region of 20%.[20,21] Partial response is often of short duration, toxicity is always severe and overall survival is poor. Use is generally confined to patients with symptomatic disease that is not amenable to surgery or radiotherapy.

In conclusion, we present the fourth and fifth cases of thyroid liposarcoma. The principles of treatment are the same as those of soft tissue sarcoma at other sites, in that complete surgical resection provides the best outcome. Narrow margins demand radical dose radiotherapy to maximise local control. The role of chemotherapy is not fully established but usually confined to the metastatic setting.

### References

1. Baloch ZW, LiVolsi VA. Newly described tumours of the thyroid. Current Diagnostic Pathology (2000) 6, 151–164 Harcourt Publishers Ltd.
2. Cook AM, Vini L, Harmer C. Squamous cell carcinoma of the thyroid gland: outcome of treatment in 16 patients. *Europ J Surg Onc* 1999; 25: 606–609.
3. Andrion A, Gaglio A, Dogliani N, Bosco E, Mazzucco G. Liposarcoma of the Thyroid Gland. Am *J Clin Path* 1991; 95: 675–679.
4. Enzinger FM, Weiss SW. Liposarcoma. In: Enzinger FM, Weiss SW, eds. *Soft tissue tumors*. St. Louis: Mosby-Year Book, 1994: 431–66.
5. Weiss S. *Histological Typing of Soft Tissue Tumours* Editors, Springer-Verlag. 1994.
6. Rosai J, Carcangiu M, Delellis R, Tumours of the Thyroid Gland. Armed Forces Inst. Of Pathology. Washington DC.
7. Suster S, Fischer C. Immunoreactivty for the human progenitor cell antigen CD34 in lipomatous tumours: *Am J Surg Path* 1997; 21: 195–200.
8. Neilsen VT, *et al.* Liposarcoma of the Thyroid Gland. *Tumori* 1986; 72: 499–502.
9. Griem KL, Robb PK, *et al.* Radiation Induced Sarcoma of the Thyroid. *Arch Otolaryngol Head Neck Surgery* 1989; 115: 8, 991–993.
10. Eeles RA, Fisher C, A'Hern RP, Robinson M, Rhys-Evans P, Henk JM, Archer D, Harmer C. Head and Neck Sarcomas: prognostic factors and implications of treatment. *Br J Cancer* 1991, 68, 201-207.
11. Harmer C, Bidmead M, Shepherd S, Sharpe A, Vini L. Radiotherapy planning techniques for thyroid Cancer. *Brit J Radiol* 1998; 71: 1069–1075.
12. Nutting CM, *et al.* Improvements in target coverage and reduced spinal cord irradiation using intensity-modulated radiotherapy (IMRT) in patients with carcinoma of the thyroid gland. *Radioth Oncol* 2001; 60: 173–180.
13. Harmer C, Bidmead M. Three-dimensional planning and conformal radiotherapy. J. Verwell, HM Pinedo and HD Suit, eds. In: *Soft Tissue Sarcomas: Present Achievements and Future Prospects*. Kluwer academic publishers, Boston 1997: 129–141.
14. O'Sullivan, *et al.* Pre-operative versus post-operative radiotherapy in soft-tissue sarcoma of the limbs: a randomised trial. *Lancet*, 2002; vol 359 (Jun29): 2235–2241.
15. Suit HD, Proppe KH, Mankin HG, Wood WC. Pre-operative radiation therapy for sarcoma of soft tissue. *Cancer* 1981; 5: 2269–2274.
16. Pollack A, Zagars G, Goswitz M, *et al.* Pre-operative versus post-operative radiotherapy in the treatment of soft tissue sarcoma: a matter of presentation. *Int J Radiat Oncol Biol Phys* 1998; 42: 563.

17. Shiu MH, Turnbull AD, *et al*. Control of locally advanced extremity soft tissue sarcomas by function saving resection and brachytherapy. *Cancer* 1984; 53: 1385–1392
18. Jacob R, Gilligan D, Robinson M, Harmer C. Hyper-fractionated radiotherapy for soft tissue sarcoma: results of the second study of hyper-fractionated radiotherapy. *Sarcoma* 1999; 3: 157–165.
19. Sarcoma meta-analysis collaboration. Adjuvant chemotherapy for localised respectable soft-tissue sarcoma of adults: meta-analysis of individual data. *Lancet* 1997; 350: 1647–54.
20. Bramwell V, Rousesse J, Steward W, *et al*. Adjuvant CyVADIC chemotherapy for adult soft tissue sarcoma—reduced local recurrence but no improvement in survival: a study of the European Organisation for Research and Treatment of Cancer, Soft tissue and Bone sarcoma Group. *J Clin Onc* 1994; 12: 1137.
21. Santoro A, Tursz T, Mourisden H, *et al*. Doxorubicin versus CyVADIC versus Doxorubicin and ifosfamide in first line treatment of advanced soft tissue sarcomas: a randomised study of the European Organisation for Research and Treatment of Cancer Soft Tissue and Bone sarcoma Group. *J Clin Oncol* 1995; 13: 1537.
22. Edmonson JH, Ryan LM, Blum RH, *et al*. Randomised comparison of doxorubicin alone versus ifosfamide plus doxorubicin or mitomycin, doxorubicin and cisplatin against advanced soft tissue sarcomas. *J Clin Oncol* 1993; 11: 1269.
